# Peripheral blood lymphocyte proviral DNA predicts neurocognitive impairment in clade C HIV

**DOI:** 10.1007/s13365-020-00882-9

**Published:** 2020-07-31

**Authors:** Vurayai Ruhanya, Graeme Brendon Jacobs, George Nyandoro, Robert H. Paul, John A. Joska, Soraya Seedat, Richard Helmuth Glashoff, Susan Engelbrecht

**Affiliations:** 1 Division of Medical Virology, Department of Pathology, Faculty of Medicine and Health Sciences, Stellenbosch University, Francie van Zijl Avenue, P.O. Box 241, Cape Town, 8000 South Africa; 2 Department of Medical Microbiology, College of Health Sciences, University of Zimbabwe, P.O. Box A178, Avondale, Harare, 00263 Zimbabwe; 3 Department of Psychological Sciences, Missouri Institute of Mental Health, University of Missouri-St. Louis, University Boulevard, St. Louis, MO USA; 4 MRC Unit on Anxiety & Stress Disorders, Department of Psychiatry & Mental Health, University of Cape Town, Cape Town, South Africa; 5 Unit on Anxiety and Stress Disorders, Department of Psychiatry, Stellenbosch University, Faculty of Medicine and Health Sciences, Stellenbosch University, Francie van Zijl Avenue, Tygerberg, 7505 South Africa; 6 Division of Medical Microbiology and Immunology, Department of Pathology, Faculty of Medicine and Health Sciences, Stellenbosch University, Francie van Zijl Avenue, Tygerberg, 7505 South Africa; 7 Division of Medical Virology, National Health Laboratory Service (NHLS), Tygerberg Business Unit, Tygerberg Hospital, Tygerberg, South Africa

**Keywords:** HIV-1 proviral DNA, HAND, Lymphocytes

## Abstract

It is not known if proviral DNA in the periphery corresponds to cognitive status in clade C as it does in clade B and recombinant forms. A cross-sectional study was conducted on participants investigated for HIV-associated neurocognitive impairment in South Africa. HIV-1 proviral DNA was quantified using a PCR assay targeting a highly conserved HIV-1 LTR-gag region. Fifty-four (36.7%) participants were cognitively impaired and 93 (63.3%) were not impaired. Forty-three (79.6%) of the cognitively impaired participants were female and 11 (20.4%) were male. There was no significant age difference between cognitively impaired and unimpaired participants (*p* = 0.42). HIV-1 DNA in cognitively impaired PLWH was significantly higher than in cognitively normal individuals (*p* = .016). Considering impaired participants, lymphocyte HIV-1 DNA was significantly higher in males than females (*p* = 0.02). There was a modest positive correlation between lymphocyte HIV-1 DNA and global deficit scores (GDS) *r* = 0.176; *p* = 0.03). The two measures of viral load, lymphocyte HIV-1 DNA copies/million and plasma RNA copies/ml, were positively correlated (r = 0.39; *p* < .001). After adjusting for other covariates, age, sex, treatment status, and the interactions between impairment and treatment, the multivariate regression showed association between proviral load and neurocognitive impairment; omega effect size was 0.04, *p* value = 0.010. The burden of HIV-1 peripheral blood lymphocyte proviral DNA corresponds to neurocognitive impairment among individuals infected with clade C disease. Therefore, therapeutic strategies to reduce the HIV-1 proviral DNA reservoir in lymphocytes may improve neurocognitive outcomes in PLWH.

## Introduction

HIV-1-associated neurocognitive disorders (HAND) remain an important clinical concern. HAND affects 40–70% of HIV-1-infected patients (Williams et al. 2014; Yusuf et al. 2017), despite immune reconstitution and viral suppression because of combination antiretroviral therapy (cART) (Cysique et al. 2015). The exact mechanism of HAND pathogenesis is not known, but it is thought to be a complex interaction of factors including cellular targets, viral factors, and the immune response (Saylor et al. 2016; Carroll and Brew 2017; Ruhanya et al. 2017). Evidence from experimental models and human studies show that HAND pathogenesis involves a seeding of peripheral HIV-1 into the central nervous system (CNS) (Price et al. 2007). HIV RNA has been detected in CSF within 8 days of infection, and immune activation is associated with neuropathogenesis (Hellmuth et al. 2015; Valcour et al. 2012). Residual viremia and HIV-1 DNA reservoirs in the periphery are linked to activation-induced neuronal damage (Cysique et al. 2015; Valcour et al. 2012) as well as chronic immune activation from macrophages, glial cells, and astrocytes (Hong and Banks 2015).

Brain invasion by HIV can develop into HIV-associated encephalitis (HIVE) which is comprised of microglial nodules, activated resident microglia, multinucleated giant cells, and infiltration by blood-derived macrophages (Williams et al. 2014; Valcour et al. 2012; Hong and Banks 2015). Clinically, people with CNS HIV-1 infection present with a spectrum of cognitive symptoms referred to as HIV-associated neurocognitive disorders (HAND) (Valcour et al. 2012). The introduction of cART has significantly reduced severe forms of HAND, although more subtle clinical presentations, which can pose difficulties for detection and monitoring, are still pervasive. Therefore, HIV-specific biomarkers are needed to assist in devising diagnostic and therapeutic approaches.

Elevated levels of HIV-1 DNA in peripheral blood mononuclear cells (PBMCs) contribute to the expression of HAND (Kamat et al. 2012; Shiramizu et al. 2009). Although evidence has demonstrated a correlation between HIV-1 DNA in PBMCs and the severity of HAND, most studies have been conducted on HIV-1 subtype B, the dominant strain in North America and Europe (Valcour et al. 2010). Data are lacking regarding HAND and subtype C, which is predominant in Southern Africa. We do not know if proviral DNA in the periphery corresponds to cognitive status in clade C as it does in clade B and recombinant forms (Valcour et al. 2013). Additionally, most studies in clade B cohorts have utilized unfractionated PBMCs and monocytes (Shiramizu et al. 2009; Kamat et al. 2012). The aim of this study was to determine whether peripheral monocyte-depleted CD14- lymphocytes correspond to HAND. We hypothesized that proviral HIV-1 DNA levels would be higher in neurocognitively impaired than in non-impaired individuals infected with HIV-1 subtype C.

## Material and methods

### Study design and patient samples

A cross-sectional study was conducted on a cohort of 147 HIV-1 positive individuals recruited from primary care HIV-1 clinics in Cape Town, South Africa. Participants were enrolled in a parent study focused on neuropsychological and brain imaging signatures in HIV-1 subtype C (Valcour et al. 2013; Paul et al. 2014).

### Neuropsychological evaluation

A battery of cognitive tests sensitive to HIV-1 was administered to all participants as described by Paul et al. 2014. Briefly, the tests were administered to assess learning, executive functions, and visuospatial and psychomotor speed. Learning was assessed as described previously (Paul et al. 2017; Valcour et al. 2013; Benedict et al. 1996). T-scores for individual cognitive tests were averaged to generate a global T-score and a global deficit score (GDS); GDS > 0.5 defined neurocognitive impairment as described elsewhere (Heaps-Woodruff et al. 2017; Jumare et al. 2017).

### Cell subset separation

Whole blood samples were collected and PBMCs separated by Ficoll gradient separation (Ficoll-Histopaque, Pharmacia, Uppsala, Sweden). CD14+ monocytes were separated by magnetic cell sorting (MACS, Miltenyi Biotec GmbH, and Bergisch Gladbach, Germany) and the peripheral blood lymphocytes (CD14-) were recovered from negative fraction of the CD14+ monocytes. The total CD3+, CD4+, and CD45+ count and percentages were determined using standardized T cell subset protocols (BDMultiset) and flow cytometry analysis (BD FACS Calibur).

### Quantification of proviral DNA in peripheral lymphocytes

HIV-1 DNA was extracted from monocyte depleted lymphocytes (CD14-) using the QIAamp Blood Mini extraction kit (Qiagen, Hilden, Germany) according to the manufacturer’s instructions. Nine specimens without complete clinical-demographic information were excluded. The quantity and purity of extracted HIV-1 DNA was determined by NanoDrop® ND-1000, (Thermo Fisher Scientific, MA, USA) spectrophotometer readings.

Quantification of total HIV-1 DNA proviral levels were determined according to the protocol described by Malnati et al. (Malnati et al. 2008), using a quantitative real-time PCR (qPCR) that targets the conserved HIV-1 LTR-*gag* region. Calibration standards for quantifying proviral DNA were made using the subtype C infectious plasmid clone, pMJ4, obtained through the NIH AIDS Reagent Program, Division of AIDS, NIAID, and NIH: (Ndung’u et al. 2001). Briefly, the concentration of the pMJ4 plasmid DNA in nanograms/microliter (ng/μl) was obtained using Nanodrop ND-1000 spectrophotometer. Plasmid DNA copy numbers per microliter (copies/μl) were then determined, using an online DNA copy calculator, on the assumption that the average weight of a base pair is 650 Da, using the following formula: mass × 6.023 × 10^23^ divided by 12,833 (length) in base pairs (bp) × 1 × 10^9^ × 660 (l Prediger 2008). From this initial concentration (copies/μl), tenfold serial dilution stocks were made, to construct a standard curve. The qPCR for the standard curve and master mix were performed using iTaq super mix (Bio-Rad, California, USA) and HIV-1 specific primers and probes, described previously by Malnati et al. 2008.

The standards were run on a CFX 96 thermocycler using Bio-Rad CFX manager 3.1 to plot the standard curve. Proviral HIV-1 DNA copies from samples were calculated from this plot using Ct (cycle threshold) values. HIV-1 cell-associated DNA (CAD) was normalized to cell input by quantification of the CCR-5 genome copies per sample. The calibration curve for quantifying genomic DNA was constructed using the CCR5 plasmid, which was obtained through the NIH AIDS Reagent Program, Division of AIDS, NIAID, and NIH: pcCCR5 (Cat#3325) (Morgenstern and Land 1990). Briefly, the concentration of CCR5 plasmid DNA was obtained using the Nanodrop ND-1000 spectrophotometer and plasmid DNA copies/μl were obtained using the online copy calculator (Prediger 2008). Sample copy numbers for genomic DNA input were then calculated from this curve, using the test sample’s Ct by the Bio-Rad CFX manager version 3.1 (Bio-Rad, California, USA).

### Statistical analysis

Data analyses were completed using Stata version 13.1 (StataCorp, College Station, Texas, USA ). HIV DNAs in lymphocytes mean differences between impaired and unimpaired were assessed using independent t test and the Cohen’s d effect size was established to quantify the extend of the difference in proviral DNA. Furthermore, covariates (age, sex, treatment status, and interaction of impairment and treatment status) were adjusted for further validation of the observed effects using multiple regression modeling. Pearson correlation coefficients assessed the association between HIV-1 peripheral blood lymphocyte DNA and neurocognitive impairment. The same test was also used to assess the association between neurocognitive impairment and clinical variables, such as plasma RNA viral load, CD4+ T cell count, monocyte count, and CD4/CD8 ratio. Statistical significance was determined as *p* value < 0.05.

## Results

### Clinical and demographic characteristics

Fifty-four (36.7%) participants were cognitively impaired and 93 (63.3%) were not impaired; thus the proportion of impaired participants was less than those not impaired (*p* = 0.0017). More females were impaired; Forty-three (79.6%) of the cognitively impaired participants were female and 11 (20.4%) were male, *p* < 0.001. There was no significant age difference between cognitively impaired and unimpaired participants (*p* = 0.42). Twenty-seven (17.8%) of the participants had initiated cART for less than 4 weeks and 125 (82.2%) had not initiated treatment, at the time of the study. The T-helper-suppressor ratio was higher in cognitively normal patients than cognitively impaired patients, although the difference was not significant (*p* = 0.33). CD14 enriched monocytes were higher in cognitively impaired participants than normal participants, although this was not statistically significant (*p* = 0.21). Table 1 summarizes the clinical and demographic characteristics of the participants in the cohort.

**Table 1:** Clinical and demographic characteristics of the cohort

	Neurocognitive status		
*Clinical & demographic variables (Mean, SD)*	*Not impaired (n = 93)*	*Impaired (n = 54)*	*p value*
Age	31.5 (5.55)	31.53 (4.70)	0.42
CD4 T-lymphocytes	243.36 (179.15)	221.56 (158.34)	0.77
CD14+	13.99 (0.62)	14.12 (0.62)	0.10
Plasma RNA viral load	96,973.29 (242238)	203,139 (545673)	0.05
CD45	1502.04 (702.03)	1515.69 (690.73)	0.45
CD4:CD8 ratio	0.31 (0.21)	0.28 (0.18)	0.33

### CD14: HIV DNA qPCR

We used a sensitive qPCR with high efficiency of more than 90% for both the normalizer CCR5 and HIV-1 with all the coefficient of determination, R^2^ greater than 0.99 for both assays. The limit of detection for the CCR5 assay was eight copies and that for HIV DNA assay was one copy per reaction.

**Fig. 1:**
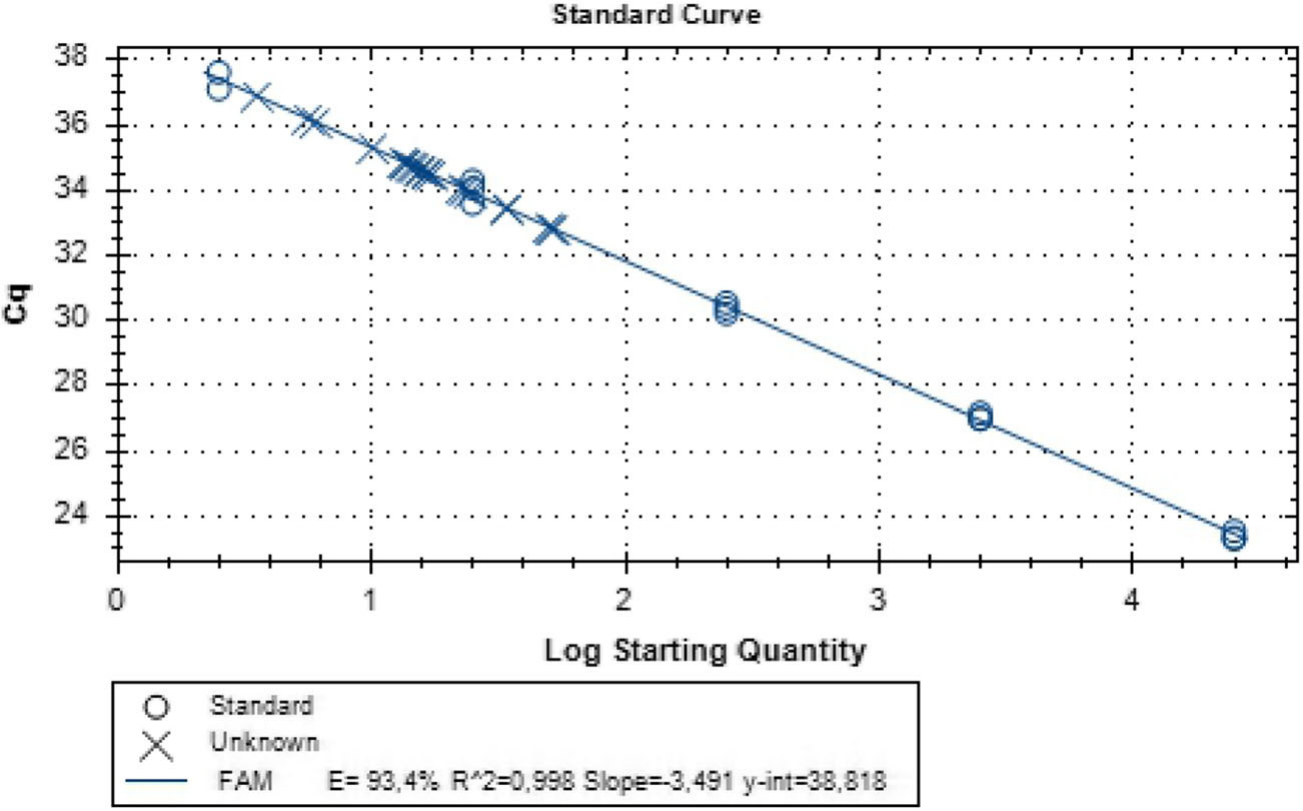
Standard curve used to estimate HIV-1 DNA copies in lymphocytes per reaction. The standard curve was generated by Bio-Rad CFX manager 3.1 using a tenfold dilution of the HIV-1 pMJ4 template, assayed in triplicate from 2.5 to 250,000 copies. Cq is plotted against the log of the starting quantity of template for each dilution. The calculated amplification efficiency was 97.4% with a slope of − 3.49 and the R^2^ value was 0.998. The y-intercept was 38.8 cycles

**Fig. 2:**
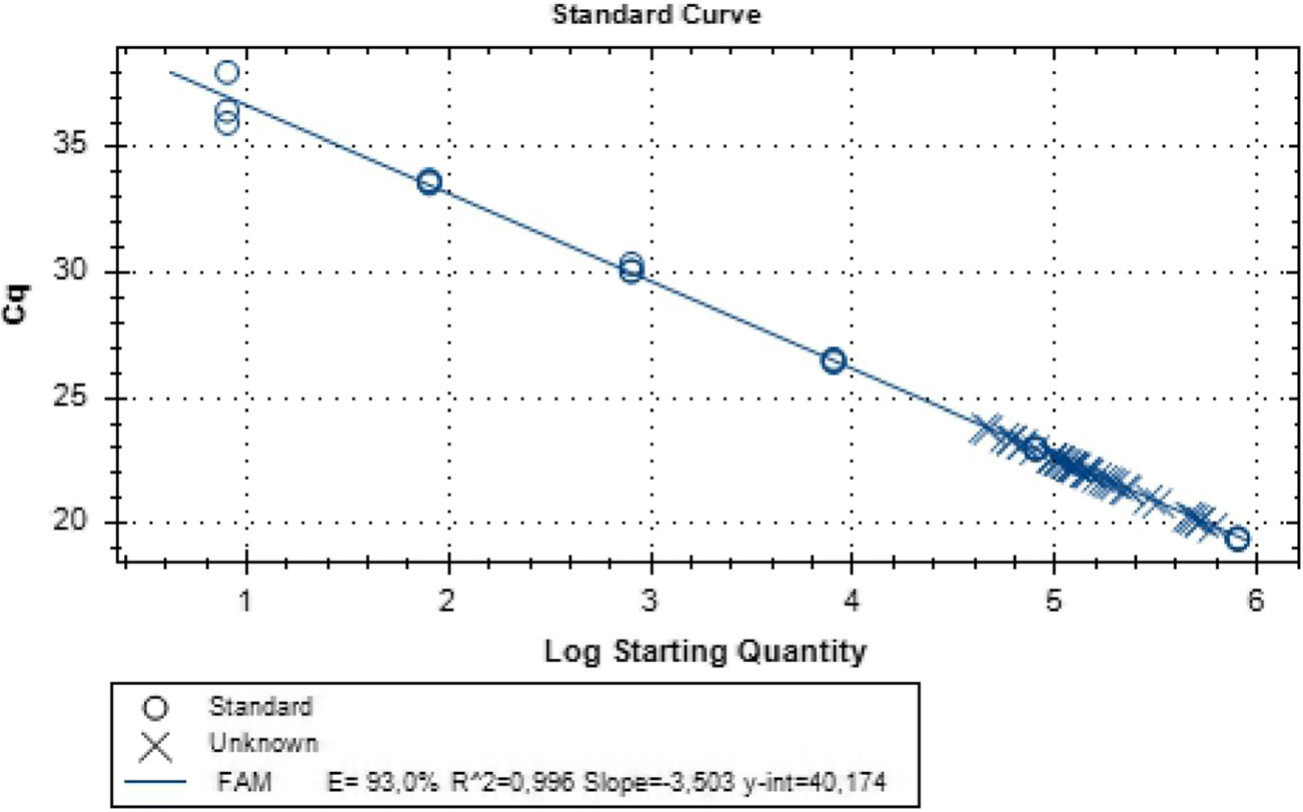
Standard curve to assess CCR5 genome copies in as cell number equivalence per reaction. The standard curve was generated by Bio-Rad CFX manager 3.1 using a 10-fold dilution of CCR5 plasmid template, assayed in triplicate from 8 to 800,000 copies. Cq is plotted against the log of the starting quantity of template for each dilution. The calculated amplification efficiency was 95.7% with a slope of − 3.429 and the R^2^ value was 1.000. The y-intercept was 40.17 cycles

Figure 1 shows standard curve for HIV starting from 3 to 2.5 × 10^4^ copies using the pMJ4 standards. The average sample input DNA quantity per reaction was 220.2 ng from which an average of 88,579 CCR5 genome copies was quantified. Figure 2 shows CCR5 standard curve with quantities ranging from 8 copies to 8 × 10^5^ copies.

The mean peripheral blood lymphocytes HIV-1 DNA for cognitively impaired PLWH was 419.76 copies per million cells compared with 240.38 in-unimpaired cases. HIV-1 DNA in cognitively impaired PLWH was significantly higher than in cognitively normal individuals (*p* = 0.016). The Cohen’s d effect size was − 0.42, lower in the unimpaired compared with impaired group with 95% confidence interval (CI): − 0.75 to − 0.08. After adjusting for other covariates, age, sex, treatment status, and the interactions between impairment and treatment, the regression omega effect size was 0.04, *p* value = 0.010; see Table 2**.**

**Table 2:** Proviral HIV-1DNA (copies/million cells) Mean differences between impaired and non-impaired groups

Group	Obs	Mean	Std. Err.	Std. Dev.	[95% Conf. Interval]	
Normal	93	240.3763	29.31945	282.7466	182.1454	298.6073
Impaired	54	419.7593	82.8949	609.1506	253.4932	586.0254
Combined	147	306.2721	36.19747	438.871	234.7334	377.8108
Diff		− 179.3829	73.85678		− 325.3578	− 33.40798
diff = mean(normal) - mean(impaired)						*t* = − 2.4288
Ho: diff = 0			Degrees of freedom		145	
Ha: diff < 0			Ha: diff! = 0		Ha: diff > 0	
Pr(T < t) = 0.0082			Pr(T > t) = 0.0164		Pr(T > t) = 0.9918	
Effect Size			Estimate	[95% Conf. Interval]		
Cohen’s d			−0 .4155383	− 0.7535504	− 0.0761216	
ttest hivdnacopiespermillion, by(sex)						
Group	Obs	Mean	Std. Err.	Std. Dev.	[95% Conf. Interval]	
Female	121	276.1901	32.75203	360.2723	211.3433	341.0368
Male	24	455.75	146.7344	718.849	152.2067	759.2933
Combined	145	305.9103	36.68911	441.7954	233.3916	378.4291
Diff		− 179.5599	97.92037		− 373.1184	13.99852
Diff = mean(female)—mean(male)						*t* = − 1.8337
Ho: diff = 0				degrees of freedom		143
Ha: diff < 0				Ha: diff! = 0		Ha: diff > 0
Pr(T < t) = 0.0344				Pr(|T| > |t|) = 0.0688		Pr(T > t) = 0.9656
Group	Obs	Mean	Std. Err.	Std. Dev.	[95% Conf. Interval]	
regress hivdnacopiespermillion age c.trt c.diag c.sex c.trt#c.diag						
Source	SS	df		MS	Number of obs =	140
					F(5, 134) = 2.81	
Model	2,644,010.25		5,528,802.051	Prob > F		= 0.0191
Residual	25,231,809.5		134,188,297.086	R-squared =		0.0948
				Adj *R*^2^ = 0.0611		
Total	27,875,819.7		139,200,545.466	Root MSE		= 433.93
HIV DNA copies per million	Coef.	Std. Err.	t	P > t	[95% Conf. Interval]	
Age	− 7.974611	7.111015	− 1.12	0.264	− 22.03896	6.089739
trt	− 77.94466	123.3087	− 0.63	0.528	− 321.8278	165.9385
Diag	219.8801	84.31013	2.61	0.010	53.1294	386.6309
Sex	163.603	100.6832	1.62	0.107	− 35.53074	362.7368
c.trt#c.diag	− 191.853	202.5405	− 0.95	0.345	− 592.4428	208.7368
_cons	322.6054	248.1464	1.30	0.196	− 168.185	813.3958
estat esize, omega						
Effect sizes for linear models						
Source	Omega-Squared	df	[95% Conf. Interval]			
Model	0.0610753	5	0		0.1363377	
Age	0.0019048	1	0		0.0583753	
trt	0	1	0		0.0395842	
Diag	0.0412042	1	0		0.1275637	
Sex	0.0120052	1	0		0.0798124	
c.trt#c.diag	0	1	0		0.051502	

**Fig. 3:**
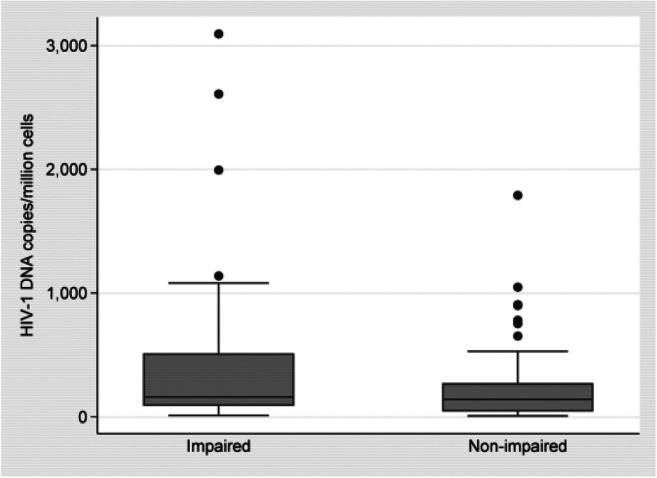
Box plots of HIV-1DNA copies per million cells by diagnosis showing significantly higher HIV-1 DNA in impaired participants than Non-impaired participants (*p* = 0.016)

Figure 3 illustrates the differences in HIV-1 DNA in impaired and unimpaired participants.

There was a modest positive correlation between lymphocyte HIV-1 DNA and global deficit scores (GDS) *r* = 0.176; *p* = 0.03). The two measures of viral load, lymphocyte HIV-1 DNA copies/million and plasma RNA copies/ml, were positively correlated (*r* = 0.39; *p* < 0.001). Considering impaired participants, proviral HIV-1 DNA was significantly higher in males than females (*p* = 0.034). There was no association between GDS and absolute CD4+ count or plasma viral load. However, there were significant differences in plasma viral load (*p* < 0.001) and CD4+ absolute count (*p* = 0.02) between cART naïve and participants initiating treatment. Other parameters (CD4:CD8 ratio, CD14+ cells, absolute CD45 count) did not differ by treatment status (*p* = 0.28, 0.1698, and 0.24, respectively). The normalizer, CCR5, did not differ by neurocognitive status (*p* = 0.21). We undertook further analysis to assess if there was an association between domain-specific cognitive impairments (e.g., learning, mental control, recall, and others that are used to calculate the GDS) and proviral HIV-1 DNA. We found that Hopkins Verbal Learning Test score (hvlt learning) (*p* = 0.049) and Wechsler Adult Intelligence Scale score (WAIS III symbol search) (*p* = 0.035) were significantly reduced with increased proviral HIV-1 DNA, when adjusted for sex, age, and treatment status. Compared with females, males had significantly lower Wechsler memory scale (WMS mental control) (*p* = 0.007), WAIS III digit symbol (*p* = 0.009), and WAIS III symbol search (*p* = 0.033) test scores; see Table 3.

**Table 3:** The effect of proviral HIV-1 DNA on cognitive subdomains

. regress hvltlearning hivdnacopiespermillion age ib0.treated ib1.Male					
hvltlearning	Coef.	Std. Err.	t	P> t	[95% Conf. Interval]
hivdnacopiespermillion	− 0.0006601	0.0003314	− 1.99	0.049	− 0.0013159 − 4.34e-06
vAge	0.0178903	0.0279508	0.64	0.523	− 0.0374236 0.0732041
Treated	0.0188188	0.3955378	0.05	0.962	− 0.7639389 .8015765
Male	− 0.2521731	0.3997102	− 0.63	0.529	− 1.043188 0.5388416
_cons	3.152604	0.9872715	3.19	0.002	1.198823 5.106385
. regress hvltrecall hivdnacopiespermillion age ib0.treated ib1.Male					
hvltrecall	Coef.	Std. Err.	t	P> t	[95% Conf. Interval]
hivdnacopiespermillion	− 0.000786	0.0003986	− 1.97	0.051	− 0.0015747 2.75e-06
age	0.0107152	0.0336163	0.32	0.750	− .0558105 0.0772409
treated	− 0.3670706	0.4757116	− 0.77	0.442	− 1.30849 .5743487
Male	.0651162	0.4807297	0.14	0.892	− .8862338 1.016466
_cons	7.304685	1.187387	6.15	0.000	4.954881 9.65449
. regress wmsmentalcontrol hivdnacopiespermillion age ib0.treated i.b1.Male					
wmsmentalcontrol	Coef.	Std. Err.	t	P > t	[95% Conf. Interval]
hivdnacopiespermillion	− 0.0020123	0.0011596	− 1.74	0.085	− 0.0043071 0.0002826
age	− 0.0521593	0.0978096	− 0.53	0.595	− 0.2457216 0.141403
treated	− 0.1953722	1.384124	− 0.14	0.888	− 2.934512 2.543768
Male	− 3.851276	1.398724	− 2.75	0.007	− 6.619311 - 1.083242
_cons		3.454805	8.37	0.000	22.08527 35.75918
. regress waisiiidigitsymbol hivdnacopiespermillion age ib0.treated i.b1Male					
waisiiidigitsymbol	Coef	Std. Err.	t	P > t	[95% Conf. Interval]
hivdnacopiespermillion	− 0.0023023	0.0025599	− 0.90	0.370	− 0.0073683 0.0027636
age	0.0760948	0.215916	0.35	0.725	− 0.3511966 0.5033861
treated	− 1.97488	3.055471	− 0.65	0.519	− 8.021569 4.071808
Male	− 8.138507	3.087703	− 2.64	0.009	− 14.24898 - 2.028034
_cons	38.19324	7.055488	5.41	0.000	24.23063 52.15584
. regress waisiiisymbolsearch hivdnacopiespermillion age ib0.treated ib1.Male					
waisiiisymbolsearch	Coef.	Std. Err.	t	P > t	[95% Conf. Interval]
hivdnacopiespermillion	− 0.0025437	0.001191	− 2.14	0.035	− 0.0049007 -0.0001868
age	− 0.0048388	0.1004548	− 0.05	0.962	− 0.2036359 0.1939583
treated	− 1.049958	1.421557	− 0.74	0.462	− 3.863176 1.763261
Male	− 3.101115	1.436552	− 2.16	0.033	− 5.944009 -0.2582203
_cons	21.3853	3.282562	6.51	0.000	14.8892 27.88139

## Discussion

This study demonstrated a significant correspondence between peripheral blood lymphocyte HIV-1 DNA load and neurocognitive impairment in adults with HIV-1 subtype C infection. This is the first study in South Africa to describe positive correlation between the monocyte-depleted fraction of peripheral blood lymphocytes and neurocognitive impairment in predominantly cART naïve HIV-1 clade C-infected participants. Peripheral blood lymphocytes, primarily the CD4+ subset, are the predominant cell type harboring HIV-1 in the blood of infected individuals (Schnittman et al. 1989; McBreen et al. 2001; Murray et al. 2014). The association of this major peripheral reservoir of HIV-1 DNA with neurocognitive impairment has significant pathological and clinical implications, particularly in the diagnosis and monitoring of the disease. Proviral DNA can be used to understand progression of HIV-associated neurocognitive impairment, particularly in subtle forms of the disease where symptomatic monitoring is difficult (Ruhanya et al. 2017). Theoretically, HIV in these cells can be targeted when devising therapeutic strategies to HIV-associated neurocognitive impairment. Infected CD4+ T lymphocytes are thought to transfect other cells, like CD8+ lymphocytes and monocytes, during the initial immune response (Gibellini et al. 2008; McBreen et al. 2001). Therefore, eradication of this reservoir might prevent the infection of peripheral monocytes, which are known to traffic HIV-1 into the brain. Infected macrophages in the brain are key factors in the development of HIV-associated neurocognitive disorders (Sasse et al. 2012).

Although the treated participants had lower lymphocyte HIV-1 proviral DNA reservoirs than treatment naïve patients, this was neither significant nor associated with improved neurocognitive performance. Previous studies found no significant reduction in proviral HIV DNA at pre- and 3-month post-treatment (Shikuma et al. 2012). Depending on the duration of cART, other findings have shown improvement in neurocognitive performance in adults on cART (Shikuma et al. 2012), but the introduction of cART has not fully eradicated neurological symptoms. Therefore, longitudinal studies are needed to examine how the reduction of peripheral lymphocyte HIV-1 DNA by cART correlates with neurocognitive performance in individuals over time. In this study, participants initiated treatment in the preceding 4 weeks; hence, the time to observe both the reduction of peripheral HIV-1 DNA and improvement in cognitive function was probably too short. It has been observed that significant reduction in peripheral HIV-1 DNA is dependent on early initiation of cART, which was not the case in this generally immunocompromised cohort (Gianella et al. 2011; Gannon et al. 2011; Watanabe et al. 2011; Herout et al. 2016).

We used a highly sensitive real-time qPCR, which is suitable for detection of very low copies (Malnati et al. 2008). It is also suitable for quantifying proviral DNA found in a relatively large amount of genomic DNA in the sample, which could interfere with detection of HIV-1 DNA. Our normalizer, CCR5 DNA copies were not different between impaired and unimpaired participants which is expected of a good normalizer (Nguewa et al. 2008). An appropriate normalizer enables true estimation of cells in the samples and accurate quantification of proviral HIV-1 DNA per sample, which reflect the quantity of the HIV reservoir, and does not vary between diseased and healthy patients.

Previous investigations have shown that total HIV-1 proviral DNA in PBMC subsets differed significantly between mild and severe forms of neurocognitive impairment, which show a gradation from very low quantities in asymptomatic to significantly higher quantities in severe forms (Valcour et al. 2013). Therefore, with regard to lymphocytes, additional studies are needed to evaluate the effects of the HIV-1 reservoir and the severity of neurocognitive impairment so that its utility as a biomarker in different stages of the disease is tested. To further strengthen the potential of lymphocyte HIV DNA role in the pathogenesis of HAND, there is also a need for association studies between lymphocyte proviral DNA quantity and biomarkers of neuronal injury and glial dysfunction (Valcour et al. 2013).

Comparison between cognitively impaired and normal participants showed that there was no statistical difference in the quantities of both CD4+ absolute count and plasma viral load. Therefore, in this study, the standard clinical markers used in routine diagnosis and clinical monitoring of HIV did not differentiate the two groups on the basis of global deficit scores. Previous studies have also demonstrated that viral load and CD4+ count are insensitive markers of HIV-1-associated disease (Clifford and Ances 2013). Levels of these two standard markers of HIV-1 infection observed in this study are in the same ranges with quantities observed in cART-naïve patients where CD4+ counts were low and plasma viral loads were relatively high (Valcour et al. 2010; Sánchez-Ramón et al. 2003).

Although the GDS construct of HAND is important, numerous published studies demonstrated that there is considerable heterogeneity in the pattern of cognitive impairment in people with HIV. We did a further analysis using domain-specific associations such as learning, mental control, recall, and others that are used to calculate GDS to assess the effect proviral HIV-1 DNA in relation to cognitive impairment. We found that Hopkins verbal learning test score (hvlt learning) and Wechsler Adult intelligence scale (WAIS III symbol search) were very significantly reduced with increased proviral HIV-1 DNA when adjusted for sex, age, and treatment status. This study demonstrates for the first time that monocyte-depleted lymphocyte proviral HIV-1DNA levels in subtype C patients is associated with neurocognitive status and specific neurocognitive domains in South Africa. We hypothesize that monocyte-depleted (CD14-) lymphocytes could be an important cellular subset housing the HIV-1 DNA and play a role in global and domain-specific neurocognitive impairment. These findings also suggest differential involvement of HIV-1 DNA in some regions of the CNS that may be leading to some cognitive domains being affected more than others.

## Conclusions

Our study showed that the burden of HIV-1 peripheral blood lymphocyte proviral DNA corresponds to neurocognitive impairment among individuals infected with clade C disease. Therefore, therapeutic strategies to reduce the HIV-1 proviral DNA reservoir in lymphocytes may improve neurocognitive outcomes in PLWH**.**

## References

[CR1] Benedict RH, Schretlen D, Groninger L, Dobraski M, Shpritz B (1996). Revision of the brief visuospatial memory test: studies of normal performance, reliability, and validity. Psycholog Assess.

[CR2] Carroll A, Brew B (2017). HIV-1-associated neurocognitive disorders: recent advances in pathogenesis, biomarkers, and treatment. F1000Research.

[CR3] Cysique LA, Hey-Cunningham WJ, Dermody N, Chan P, Brew BJ, Koelsch KK (2015). Peripheral blood mononuclear cells HIV-1 DNA levels impact intermittently on neurocognition. PLoS One.

[CR4] Gannon P, Muhammad Z, Kolso K, Kolso DL (2011). Current understanding of HIV-1-associated neurocognitive disorders pathogenesis. Curr Opin Neurol.

[CR6] Gianella S, Wyl V, Fischer M, Niederoest B, Battegay M, Bernasconi E, Cavassini M, Rauch A, Hirschel B (2011). Effect of early antiretroviral therapy primary HIV-1infection on cell-associated HIV-1DNA and plasma HIV-1infection. Antivir Ther.

[CR7] Gibellini D, Borderi M, De Crignis E, Cicola R, Cimatti L, Vitone F, Chiodo F, Re MC (2008). HIV-1DNA in peripheral blood monocytes and lymphocytes from naïve and HAART-treated indviduals. J Inf Secur.

[CR8] Heaps-Woodruff JM, Joska J, Cabeen R, Baker LM, Salminen LE, Hoare J, Laidlaw DH, Wamser-Nanney R, Peng CZ, Engelbrecht S, Seedat S, Stein DJ, Paul RH (2017). White matter fiber bundle lengths are shorter in cART naive HIV: an analysis of quantitative diffusion tractography in South Africa. Brain Imaging Behav.

[CR9] Hellmuth J, Valcour V, Spudich S (2015). CNS reservoirs for HIV: implications for eradication. J Virus Erad.

[CR10] Herout S, Mandorfer M, Breitenecker F, Reiberger T, Grabmeier-Pfistershammer K, Rieger A (2016). Impact of early initiation of antiretroviral therapy in patients with acute HIV-1 infection in Vienna, Austria. PLoS One.

[CR11] Hong S, Banks WA (2015). Role of immune system in neuroinflammation and neurocognitive implications. Brain Behav Immun.

[CR12] Jumare J, Sunshine S, Ahmed H, El-Kamary SS, Magder L, Hungerford L (2017). Peripheral blood lymphocyte HIV-1 DNA levels correlate with HIV-1 associated neurocognitive disorders in Nigeria. J Neuro-Oncol.

[CR13] Kamat A, Misra V, Cassol E, Ancuta P, Yan Z (2012). A plasma biomarker signature of immune activation in HIV patients on antiretroviral therapy. PLoS One.

[CR14] Malnati MS, Scarlatti G, Gatto F, Salvatori F, Cassina G, Rutigliano T, Volpi R, Lusso P (2008). A universal real-time PCR assay for the quantification of group-M HIV-1 proviral load. Nat Protoc.

[CR15] McBreen S, Imlach S, Shirafuji T, Scott GR, Leen C, Bell JE, Simmonds P (2001). Infection of the CD45RA1 (naive) subset of peripheral CD81 lymphocytes by human immunodeficiency virus type 1 in vivo. J.Virol.

[CR16] Morgenstern JP, Land H (1990). Advanced mammalian gene transfer: high titre retroviral vectors with multiple drug selection markers and a complementary helper-free packaging cell line. Nucleic Acids Res.

[CR17] Murray JM, Zaunders JJ, McBride KL, Xu Y, Bailey M, Suzuki K, Cooper DA, Emery S, Kelleher AD, Koelsch KK (2014). HIV-1 DNA subspecies persist in both activated and resting memory CD4−T cells during antiretroviral therapy. J Virol.

[CR18] Ndung’u T, Renjifo B, Essex M (2001). Construction and analysis of an infectious human immunodeficiency virus type 1 subtype C molecular clone. J Virol.

[CR19] Nguewa PA, Agorreta J, Blanco D, Lozano MD, Gomez-Roman J, Sanchez BA (2008). Identification of importin 8 (IPO8) as the most accurate reference gene for the clinicopathological analysis of lung specimens. BMC Mol Biol.

[CR20] Paul RH, Joska JA, Woods C, Seedat S, Engelbrecht S, Hoare J (2014). Impact of the HIV-1 tat C30C31S dicysteine substitution on neuropsychological function in patients with clade C disease. J Neuro-Oncol.

[CR21] Paul RH, Phillips S, Hoare J, Laidlaw DH, Cabeen R, Olbricht GR, Su Y (2017). Neuroimaging abnormalities in clade C HIV are independent of tat genetic diversity of tat genetic diversity. J Neuro-Oncol.

[CR22] Prediger E (2008) Calculations: converting from nanograms to copy number. S://www.idtdna.com/pages/education/decoded/article/calculations-converting-from-nanograms-to-copy-number accessed 13/09/2018

[CR23] Price RW, Epstein LG, Becker JT, Cinque P, Gisslen M, Pulliam L, McArthur JC (2007). Biomarkers of HIV-1CNS infection and injury. Neurology.

[CR27] Ruhanya V, Jacobs GB, Glashoff RH, Engelbrecht S (2017). Clinical relevance of Total HIV-1 DNA in peripheral blood mononuclear cell compartments as a biomarker of HIV-1-associated neurocognitive disorders (HAND). Viruses.

[CR28] Sánchez-Ramón S, Bellón JM, Resino S, Cantó-Nogués C, Gurbindo D, Ramos JT, Muñoz-Fernández AM (2003). Low blood CD8+ T-lymphocytes and high circulating monocytes are predictors of HIV-1-1-associated progressive encephalopathy in children. Pediatrics..

[CR29] Sasse T, Wu J, Zhou L, Saksena K (2012). Monocytes and their role in human immunodeficiency virus pathogenesis. AJDM..

[CR30] Saylor D, Dickens AM, Sacktor N, Haughey N, Slusher B, Pletnikov M, Mankowski JL, Brown A, Volsky DJ, McArthur JC (2016). HIV-1-associated neurocognitive disorder — pathogenesis and prospects for treatment. Nat Rev Neurol.

[CR31] Schnittman SM, Psallidopoulos MC, Lane HC, Thompson L, Baseler M, Massari F, Fox CH, Salzman NP, Fauci AS (1989). The reservoir for HIV-1in human peripheral blood is a T cell that maintains expression of CD4. Science.

[CR32] Shikuma CM, Nakamoto B, Shiramizu B, Liang CY, DeGruttola V, Bennett K, Paul R, Kallianpur K, Chow D, Gavegnano C, Hurwitz SJ, Schinazi RF, Valcour VG (2012). Antiretroviral monocyte efficacy score linked to cognitive impairment in HIV-1. Antivir Ther.

[CR34] Shiramizu B, William S, Shikuma CM, Valcour V (2009). Amount of HIV-1 DNA in peripheral blood mononuclear cells is proportional to the severity of HIV-1-1-associated neurocognitive disorders. J Neuropsychiatry Clin Neurosci.

[CR35] Valcour VG, Shiramizu BT, Shikuma CM (2010). HIV-1 DNA in circulating monocytes as a mechanism to dementia and other HIV-1 complications. JLB..

[CR36] Valcour V, Chalermchai T, Sailasuta N (2012). Central nervous system viral invasion and inflammation during acute HIV infection. J Infect Dis.

[CR37] Valcour VG, Ananworanich J, Agsalda M, Sailasuta N, Chalermchai T, Schuetz A (2013) HIV-1 DNA reservoir increases risk for cognitive disorders in cART-naïve patients. PLoS One. 10.1371/journal.pone.0070164 10.1371/journal.pone.0070164 PMC3729685 23936155

[CR38] Watanabe D, Ibe S, Uehira T, Minami R, Sasakawa A, Yajima K, Yonemoto H, Bando H, Ogawa Y, Taniguchi T, Kasai D, Nishida Y, Yamamoto M, Kaneda T, Shirasaka T (2011). Cellular HIV-1DNA levels in patients receiving antiretroviral therapy strongly correlate with therapy initiation timing but not with therapy duration. BMC Infect Dis.

[CR39] Williams DW, Veenstra M, Gaskill PJ, Morgello S, Calderon TM, Berman JW (2014). Monocyte mediate HIV-1 neuropathogenesis: mechanisms that contribute to HIV-1 associated neurocognitive disorders. Curr HIV-1 Res.

[CR40] Yusuf AJ, Hassan A, Mamman AI, Muktar HM, Sulieman AM, Baiyewu O (2017). Prevalence of HIV-1-associated neurocognitive disorder (HAND) among patients attending a tertiary health facility in Northern Nigeria. J Int Assoc Provid AIDS Care.

